# Interventions for mental health, cognition, and psychological wellbeing in long COVID: a systematic review of registered trials

**DOI:** 10.1017/S0033291722002203

**Published:** 2022-06-30

**Authors:** Lisa D. Hawke, Anh T. P. Nguyen, Chantal F. Ski, David R. Thompson, Clement Ma, David Castle

**Affiliations:** 1 Centre for Complex Interventions, Centre for Addiction and Mental Health, Toronto, ON, Canada; 2 Department of Psychiatry, University of Toronto, Toronto, ON, Canada; 3 Integrated Care Academy, University of Suffolk, Ipswich, UK; 4 School of Nursing and Midwifery, Queen's University Belfast, Belfast, UK

**Keywords:** COVID-19, mental health, psychosocial interventions, systematic review protocol, wellbeing

## Abstract

**Background:**

Among patients diagnosed with COVID-19, a substantial proportion are experiencing ongoing symptoms for months after infection, known as ‘long COVID’. Long COVID is associated with a wide range of physical and neuropsychological symptoms, including impacts on mental health, cognition, and psychological wellbeing. However, intervention research is only beginning to emerge. This systematic review synthesizes currently registered trials examining interventions for mental health, cognition, and psychological wellbeing in patients with long COVID.

**Methods:**

Standard systematic review guidelines were followed. Trials registered in two large trial registries in 2020 to May 2022 were reviewed. Included studies were narratively synthesized by type of intervention and a risk-of-bias assessment was conducted.

**Results:**

Forty-two registered trials were included, with a total target sample size of 5814 participants. These include 11 psychological interventions, five pharmacological and other medical interventions, and five evaluating herbal, nutritional, or natural supplement interventions. An additional nine trials are examining cognitive and neurorehabilitation interventions and 12 are examining physiotherapy or physical rehabilitation. Most trials are randomized, but many are feasibility trials; trials are evaluating a wide spectrum of outcomes.

**Conclusions:**

While there is a newly emerging body of research testing interventions for mental health, cognition, and psychological wellbeing in long COVID, the breadth and scope of the research remains limited. It is urgently incumbent on researchers to expand upon the intervention research currently under way, in order to generate high-quality evidence on a wide range of candidate interventions for diverse long COVID patient populations.

While COVID-19 infection is usually associated with a brief illness followed by recovery within weeks, many people experience prolonged symptoms months after acute infection (Liu et al., 2021a). Prolonged symptoms after an acute COVID-19 infection have been given a number of names, including long COVID, post-COVID syndrome, and COVID long haulers (Raveendran, Jayadevan, & Sashidharan, 2021). Long COVID is associated with a wide variety of symptoms, including fatigue, headaches, shortness of breath, loss of sense of smell, ‘brain fog’, cognitive impairment, neuropathy, muscle pain, sleep disturbance, and other multi-system symptoms, along with reduced mental health and impaired quality of life (Crook, Raza, Nowell, Young, & Edison, 2021; Malik et al., 2022; Maury, Lyoubi, Peiffer-Smadja, de Broucker, & Meppiel, 2020; Sudre et al., 2021).

A meta-analytic estimate of the pooled prevalence of long COVID indicates that, around the world, some 43% of those who contract COVID-19 experience long-term symptoms (Chen et al., 2022). Risk factors include female sex, pre-existing asthma, older age, obesity, comorbidities, and more severe acute COVID-19 symptoms (Cabrera Martimbianco, Pacheco, Bagattini, & Riera, 2021; Chen et al., 2022; Sudre et al., 2021). Social isolation, decreased physical activity, changed lifestyles, and pandemic-related social and economic insecurities may contribute to developing the physical and psychological symptoms of long COVID (Cabrera Martimbianco et al., 2021; Marshall, Bibby, & Abbs, 2020). For some, long COVID may become a protracted, debilitating, multi-systemic disability (Alwan, 2021; Brown & O'Brien, 2021).

The COVID-19 pandemic has had substantial mental health repercussions (Jenkins et al., 2021a), as the public health restrictions that aim to reduce the spread of the virus have disrupted many protective factors for mental health and wellness (Heinsch et al., 2022; Hoare, Milton, Foster, & Allender, 2016; Silva, Loureiro, & Cardoso, 2016). Depression, anxiety, and distress have increased among the general population during the pandemic (Aknin et al., 2022), as social interaction, pro-social activities, physical activity, and everyday life have been radically transformed. The research on mental health in long COVID remains scant (Vannorsdall & Oh, 2021). However, it appears that long COVID can be accompanied by anxiety, depression, and post-traumatic stress disorder, as well as neurocognitive issues (Raveendran et al., 2021); these, in turn, can be complicated by the physiological and neurological symptoms that prevent people from returning to their previous level of functioning (Aiyegbusi et al., 2021). People with long COVID are at risk of combining the mental health impacts of long COVID with those associated with population-level pandemic response strategies (Brüssow & Timmis, 2021). Unfortunately, as mental healthcare needs have increased, the mental healthcare system has also been disrupted (Mann, Chen, Chunara, Testa, & Nov, 2020). A shift to virtual care has exacerbated the digital divide and changed willingness to seek services (Hoyer et al., 2021), compromising access to timely mental health support.

The National Institute for Health and Care Excellence (NICE) has issued clinical practice guidelines for the treatment of long COVID, in which they recommend integrated and interdisciplinary models of care to meet the wide range of long-term needs with which these individuals may present (International Foundation of Integrated Care, 2020). As part of integrated treatment models, it is critical that we combine physical healthcare with social services, mental health supports, cognitive rehabilitation, and psychiatric treatments when indicated (Aiyegbusi et al., 2021). Given the complexity of the physical, cognitive, psychological, and social impacts of long COVID in the context of the ongoing pandemic, there is a need for multi-facetted, complex interventions that are adapted to the individual and the local context. This intervention complexity requires appropriate evaluation, ideally following the Medical Research Council's framework for evaluating complex interventions (Skivington et al., 2021).

Interventions that support mental health and psychological wellbeing have been shown to help people with physical health challenges (Ferrier et al., 2021; Gilbert et al., 2012; Jenkins et al., 2021b). By building resiliency in vulnerable populations, it is possible to build positive mental health that supports disease management and improves quality of life. Pharmacological interventions are also sometimes indicated for mental health problems secondary to physical health diagnoses (National Collaborating Centre for Mental Health, 2010). It is therefore important to consider such interventions within multi-component, integrated models of care for long COVID. However, since long COVID is an emerging clinical entity, effective interventions for this complex condition constitute a critical gap in the literature. To advance research agendas in this area, it is important to understand the current state of the research, even at this early stage, in the absence of a large body of published evidence.

This systematic review aims to support the rigorous planning of research agendas by synthesizing the currently registered trials examining interventions for mental health, cognition, and psychological wellbeing in long COVID.

## Methods and analysis

This systematic review of registered trials follows the Preferred Reporting Items for Systematic Reviews and Meta Analyses (PRISMA) (Page et al., 2021).

### Trial retrieval

An electronic search was conducted of two large, international registries for clinical trials: clinicaltrials.gov, a trial registry by the United States National Library of Medicine, and the International Clinical Trials Registry Platform, an aggregator of international trial registries by the World Health Organization. The search was conducted on 4 January 2022 and updated on 31 May 2022, covering the first 5 months of 2022.

Recognizing that this is a new, emerging literature, multiple search strategies were piloted to identify keywords. Based on the relatively small number of trials on the topic, it was decided not to use intersecting search terms, to balance risk of missing trials *v.* over-screening. Therefore, search terms were long COVID, OR post COVID, OR post-acute COVID, OR long haul*, OR COVID sequelae, OR sequelae of COVID, OR COVID survivor, OR post-SARS-COV-2. There were two filters: (1) the date-of-registration of the trial had to be between 1 January 2020 and 31 December 2021 or between 1 January 2022 and 31 May 2022 for the update; (2) only interventional trials were included (filter available on clinicaltrials.gov only). All trials found with these search parameters were uploaded into Covidence systematic review software (Veritas Health Innovation, 2021), where duplicates were automatically deleted and visually confirmed. For records in the final record set, published articles with outcome data were manually sought in MEDLINE and PsycINFO using the study ID and lead researcher names.

### Eligibility criteria

From a PICO (population, intervention, control, outcomes) perspective (Richardson, Wilson, Nishikawa, & Hayward, 1995), studies were eligible if they addressed individuals with long COVID, used any intervention, with or without a comparison group, and had either a primary intervention aim or primary outcome specific to mental health, cognition, or psychological wellbeing. Studies with quality of life as a primary outcome were included only if the quality of life measure contained a mental health or psychological wellness subscale, ensuring that a mental/psychological component is to be included in the outcomes rather than limiting the outcome to physical health-related quality of life. Each study's own definition of long COVID was accepted, provided that the record referred to the concept of long COVID and recruited individuals at a minimum of 1 month after acute COVID-19. Records could originate from any country and could report on participants of any age group and with any sociodemographic characteristics. Excluded were trials registered prior to 2020 (i.e. before the pandemic) or after 31 May 2022, non-interventional trials, and trials that did not focus on mental health, cognition, or psychological wellbeing.

### Study selection

The initial search yielded a total of 912 records, among which 150 duplicates were automatically removed by Covidence (Veritas Health Innovation, 2021), for an initial search of 762 records. The update produced an additional 158 records, including 18 duplicates, for a total of 902 records. These records were reviewed first at the title and project summary level based on inclusion and exclusion criteria. After a training and calibration review of 25 records by a research lead and a research staff, the research staff and lead both independently screened 152 records (152/762 = 20%), achieving 92.8% agreement, with an inter-rater agreement of *κ* = 0.74 [substantial agreement (Sim & Wright, 2005)]. Any conflicts were resolved by consensus. The research staff independently screened the remaining records, with open discussion of any uncertainties. An additional 30 duplicates were removed manually during screening. Records that were retained were then screened at the full record level by both the research lead and research staff for 21 initial records (21/100 = 21%), with 90.5% agreement [*κ* = 0.74, substantial agreement (Sim & Wright, 2005)]. The research staff screened the remaining records at the initial search and all records at the updated search independently, bringing any uncertainties to the project lead for discussion and resolution by consensus. The final record set was reviewed and confirmed by the research lead.

### Data extraction and synthesis

Data were extracted into an Excel spreadsheet by the study staff, with ongoing discussion. Data extraction included descriptive information about the (1) trial as a whole (i.e. the study ID, funder/sponsor, date of registration, country of the principal investigator, countries of recruitment, scientific title), (2) intervention (i.e. name, type, description, delivery mode, dose, frequency, length), (3) study design (i.e. allocation, model, masking, arms, recruitment status, start, and expected end date), (4) sample (i.e. long COVID definition if available, age, sample size, inclusion, exclusion criteria), and (5) outcome measures (i.e. primary outcome(s), secondary outcome(s), measures, timing of measurement). Data were synthesized and summarized in narrative and table format based on the type of intervention.

### Quality assessment

The Cochrane Risk of Bias 2.0 (Higgins, Savović, Page, Elbers, & Sterne, 2021) guidelines were used to examine study quality. Since the review was conducted on registered trials rather than publications presenting outcomes, only minimal variables were available. Of the five domains in the Cochrane guidelines, domain 1 (randomization process) was partially reviewed for randomization and allocation concealment; domain 2 (deviations from the intended intervention) was partially reviewed for participant and care provider masking only; domain 3 (missing outcome data) was not reviewed, since no outcome data are available; domain 4 (outcome measurement process) was fully reviewed. Domain 5 (reported results) was not reviewed, since no results have been reported. A partial risk-of-bias determination was made, based on the retained domains. The risk-of-bias assessment was conducted collaboratively, with the study lead and a study staff member completing 11/28 (39.3%) of the initial studies together, and the staff member completing the remaining alone, bringing any questions forward for discussion. Results of the partial risk-of-bias assessment are narratively reported.

## Results

**Fig. 1.**
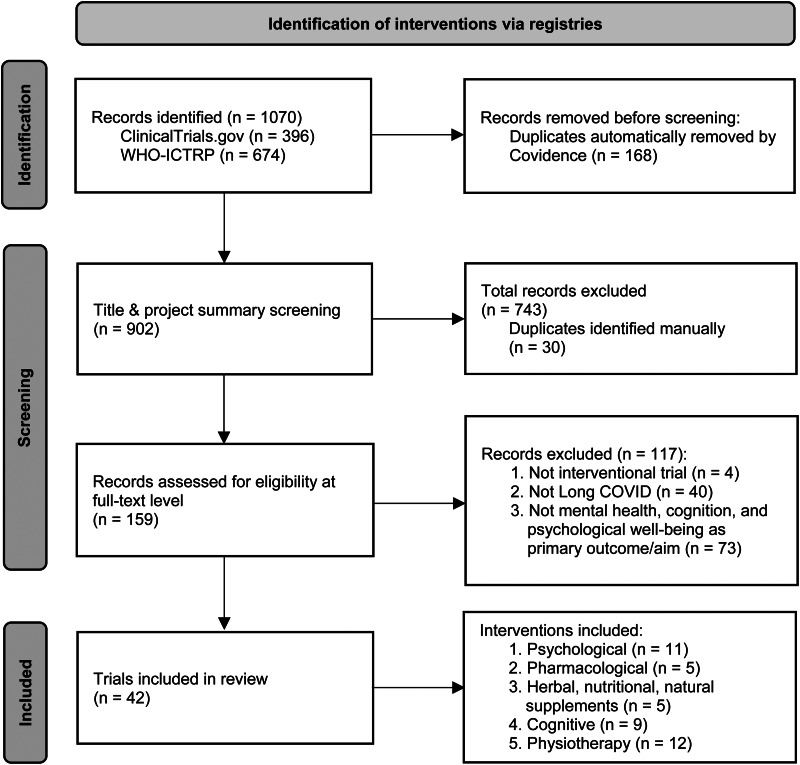
PRISMA flow chart for trials identified in the systematic review.

Of a total of 902 identified records, 42 were eligible for inclusion; see the PRISMA diagram in Fig. 1 (Page et al., 2021). Associated with the selected trials, one published protocol was found (Gao et al., 2021); publications presenting outcome data were not available for any of the trials. General trial characteristics are described in Table 1 and detailed trial information is provided in Table 2. The 42 records report on trials of psychological interventions, cognitive or neurorehabilitation interventions, pharmacological and other medical interventions; herbal, nutritional, or natural supplements; and physiotherapy or physical rehabilitation interventions.

**Table 1.** Characteristics of the 42 trials included in the systematic review

	*n*	%
Intervention type		
Psychological	11	26.2
Cognitive and neurorehabilitation	9	21.4
Herbal, nutritional, natural supplement	5	11.9
Pharmacological and other medical	5	11.9
Physiotherapy and physical rehabilitation	12	28.6
Allocation		
Randomized efficacy	23	54.8
Randomized feasibility/pilot	12	28.6
Non-randomized	7	16.7
Age range		
<18	0	0.0
18–64	42	100.0
65+	31	73.8
Virtual/remote delivery	22	52.4
Location		
North America	20	47.6
Europe	17	40.5
Asia	2	4.8
South America	2	4.8
Australia	1	2.4
Recruitment status		
Not yet recruiting	13	31.0
Recruiting	27	64.3
Completed	2	4.8
Expected study completion (*n* = 21, one missing)		
2021	3	7.1
2022	18	42.9
2023	14	33.3
2024	5	11.9
2025	1	2.4
Primary outcome		
Depression, anxiety, stress	9	21.4
Cognitive functioning	18	42.9
Psychological well-being	17	40.5

**Table 2.** Complete summary of the 42 trials included in the review

Study ID and reference	Intervention title	Intervention summary	Country	Study period (m/y-m/y)	Design	Control group	Target N	Intervention dose/duration	Primary outcomes	Secondary outcomes	Assessment times	Risk of bias
Psychological interventions												
ISRCTN-38746119 (Chantal et al., 2021)	Long COVID optimal health program (LC-OHP)	Self-efficacy and disease management intervention	United Kingdom	09/21–10/22	Feasibility/pilot RCT, single-blinded	TAU	60	1 h individual or 90 min group sessions, weekly, 5 weeks	Feasibility, acceptability	Depression, anxiety, self-efficacy, QOL, fatigue	Baseline, 3 m, 6 m post randomization	High risk^a^
NCT-05139979 (Subramaniam, 2021)	Yogic breathing and guided meditation for long COVID symptoms	Breathing and meditation exercise	United States	09/21–12/23	Feasibility/pilot RCT, open label	Waitlist	68	3 daily webinars, 3 weeks	Compliance	Stress, mood, QOL, dyspnea, somatic symptoms	Baseline, week 1, 2, 3	High risk^a^
NCT-04949061 (Acartürk & Öztürk, 2021)	The effectiveness of culturally adapted cognitive behavioral intervention among COVID-19 survivors	CBT with mindfulness, meditation, applied stretching	Turkey	11/21–12/22	RCT, open label	Enhanced TAU	86	1 weekly session, 8 weeks	Psychological distress	Depression, anxiety, PTSD, somatic complaints, QOL, psychological flexibility, emotion regulation	Baseline, week 1, 5 weeks post intervention	High risk
NCT-05119608 (Håkansson et al., 2021)	Treatment of post-COVID syndrome in patients treated in intensive care	CBT with principal components of ACT	Sweden	11/21–11/25	Feasibility/pilot RCT, open label	TAU	128	1 weekly session, 10 weeks	Anxiety	Depression, QOL, fatigue, PTSD, patient satisfaction	Baseline, week 2, 3 m, 12 m post treatment	High risk^a^
NCT-05107440 (Culos-Reed & Twomey, 2021)	BREATHE: virtual self-management for long COVID-19	Psychoeducation on breathing, rest/recovery, other health behaviors	Canada	10/21–10/22	Feasibility/pilot non-RCT, open label	NA	36	2 weekly sessions for 8 weeks	Self-efficacy, daily activities, emotions	Fatigue, breathlessness, functional outcomes, QOL, attendance, safety, tolerability	Baseline, week 9, 3 m	High risk^a^
NCT-05019963 (Hatcher et al., 2021)	Enhancing COVID rehabilitation with technology (ECORT)	Electronic case management platform together with usual care	Canada	11/21–08/22	RCT, open label	TAU	152	Daily, 3 months	Health and disability (cognition subscale)	Long COVID symptoms, depression, anxiety, sleep, PTSD, QOL, fatigue, pain, breathlessness mental wellbeing, cognitive functioning	Baseline, week 4, week 8, week 12	High risk
NCT05167266 (Collette et al., 2022)	Long-COVID: treatment of cognitive difficulties	Psychoeducation	Belgium	03/22–06/23	RCT, double-blinded	N/A	130	Weekly 90 min individual sessions, 4 weeks	Subjective cognitive difficulties (behavioral regulation, metacognition)	QOL, work productivity, activity impairment, fatigue level, sleep quality, psychological distress	Baseline, 2 m, 8 m post intervention	High risk
NCT05199233 (Croghan et al., 2022)	Mindfulness intervention for post-COVID symptoms	Mindfulness meditation with a wearable headband-style device	United States	06/22–12/23	Non-RCT, open label	N/A	60	10-min, 4 times/week, 12 weeks	Stress, anxiety	N/A	Baseline, 3 m	High risk
ISRCTN11868601 (Martin & Lynall, 2022)	Development and non-randomized controlled trial of the Hope program for people living with long COVID	Peer support and self-management program	United Kingdom	07/21–04/22	Feasibility/pilot, non-RCT, open label	Waitlist	94	8 weeks	Positive mental wellbeing	Self-efficacy, fatigue, loneliness depression, anxiety, long COVID symptoms	Baseline, week 8	High risk^a^
ISRCTN91104012 (Blázquez, 2022)	Analysis of the symptoms and quality of life of people with a diagnosis of long COVID-19, and the effectiveness of an intervention in primary care	Multimodal hybrid program (app-based and face-to-face) combining cognitive exercises, physical activity, respiratory exercises, Mediterranean diet, sleep hygiene, social activation, community resources, motivational interviewing, supervision	Spain	01/22–12/24	RCT, single-blinded	TAU	95	30 min weekly, 3 weeks	QOL	Cognitive functioning, respiratory status, physical activity, adherence to diet, sleep, depression, anxiety, social support, self-efficacy, patient activation in their own health, use of health and social services	Baseline, 3 m, 6 m, and 12 m	High risk
ISRCTN36407216 (Busse & Potter, 2022)	Effectiveness and cost-effectiveness of a personalized self-management support intervention for non-hospitalized people living with long COVID	Self-management resource and individual coaching	United Kingdom	08/21–07/23	RCT, open label	TAU	558	6 sessions, 10 weeks	Routine activities	Emotional well-being, social engagement, QOL, fatigue, healthcare resource use, the cost-effectiveness, program evaluation	Baseline, 3 m post randomization	High risk
Pharmacological and other medical interventions												
NCT-05047952 (McIntyre & Subramaniapillai, 2021)	Vortioxetine for post-COVID-19 syndrome	Vortioxetine	Canada	09/21–09/22	RCT, quadruple-blinded	Placebo	200	10–20 mg/day, 8 weeks	Cognitive functioning	Cognitive functioning, depression, anxiety, QOL, emotional and physical wellbeing	Baseline, week 8	Some concern
NCT-04705831 (Melamed et al., 2021)	Study to evaluate the benefit of RUCONEST in improving neurological symptoms in post COVID-19 infection	Ruconest	United States	12/20–01/22	RCT, double-blinded	Placebo	40	Weekly infusion, 16 weeks	Cognitive functioning, depression, QOL, daily living, physical health parameters	N/A	Baseline, week 5, week 9, week 14, week 17	High risk
NCT-04904536 (Anderson & Carcel, 2021)	Statin treatment for COVID-19 to optimize neurological recovery	Atorvastatin	Australia	10/21–07/23	RCT, single-blinded	TAU	400	40 mg/day for 6 months	Cognitive functioning	Brain imaging	Baseline, 18 m	High risk
NCT05212831 (Glezer, 2022)	Portable oxygen concentrator (POC) *v.* standard of care in long COVID: randomized crossover exploratory pilot study	Supplemental oxygen using a portable oxygen concentrator (POC)	United States	04/22–12/22	Feasibility/pilot RCT, open label	TAU	20	3 h daily, 2 weeks	Pulmonary functioning, cognitive functioning	Functional status, anxiety, mood, subjective cognitive impairment, pulmonary functioning, safety outcomes	Baseline, 14 ± 3 days	High risk^a^
NCT05346120 (Alderman et al., 2022)	Post-acute COVID-19, inflammation, and depression	Stem cell infusion with allogeneic marrow stromal cells (MSCs)	United States	05/22–05/24	RCT, double-blinded	Placebo	32	One time, 30 min	Depression	QOL fatigue, pain, anger, anxiety	Baseline, day 90	Some concern
Herbal, nutritional, or natural supplement interventions												
NCT-04997395 (Iveson et al., 2021)	Feasibility of cannabidiol for the treatment of long COVID	Full spectrum cannabidiol-dominant medicinal cannabis (MediCabilis CBD)	United Kingdom	09/21–09/22	Feasibility/pilot non-RCT, open label	N/A	30	N/A	Recruitment rate, tolerability, side effects	Long COVID symptoms, fatigue, QOL, pain, mood/anxiety, sleep, physical health parameters	Monthly	High risk^a^
ISRCTN-12595520 (Blane et al., 2021)	Does weight management improve long COVID symptoms in people with long COVID and obesity?	Counterweight Plus/DiRECT diet	United Kingdom	03/21–11/23	Feasibility/pilot RCT, open label	Waitlist	200	Daily, 6 months	Fatigue, dyspnea, pain, anxiety, depression, other health symptoms	Long COVID symptoms, QOL, work productivity, weight, acceptability, barriers, costs	Baseline, 3 m, 6 m	High risk^a^
NCT-04809974 (Guzman-Velez et al., 2021)	Clinical trial of niagen to examine recovery in people with persistent cognitive and physical symptoms after COVID-19 illness (long COVID)	Nicotinamide Riboside (vitamin B3)	United States	07/21–12/22	RCT, quadruple-blinded	Placebo	100	2000 mg daily, 22 weeks	Cognitive functioning	Depression, anxiety, long COVID symptoms	Baseline, every 5 weeks for 22 weeks	Some concern
NCT-05104749 (Rice & Jacobs, 2021)	Homeopathic treatment of post-acute COVID-19 syndrome	Homeopathic medicine	United States	09/21–02/22	Feasibility/pilot RCT, quadruple-blinded	Placebo	62	N/A	Fatigue, QOL	General health	Baseline, week 4, week 8, week 12	Some concern^a^
NCT-04795557 (Karosanidze & Panossian, 2021)	Efficacy of adaptogens in patients with long COVID-19	ADAPT-232: *Rhodiola rosea* roots, *Schisandra chinensis* berry, *Eleutherococcus senticosus* root	Georgia	04/21–12/21	RCT, quadruple-blinded	Placebo	100	30 ml, 2x/day, 2 weeks	Duration and severity of long COVID symptoms, recovery, home stay	Multiple physical health parameters, cognitive functioning, anxiety and depression	Baseline, day 14, day 21	Some concern
Cognitive and neurorehabilitation interventions												
NCT-05092516 (Eryilmaz et al., 2021)	Home-based brain stimulation treatment for post-acute sequelae of COVID-19 (PASC)	Home-based transcranial direct current stimulation (tDCS)	United States	02/22–07/23	RCT, double-blinded	Placebo	40	2 mA of anodal stimulation, 30 min daily, 4 weeks	Cognitive functioning	N/A	Baseline, week 4, week 8	High risk
RBR-77jbq56 (Neri & Barcessat, 2021)	Evaluation of the use of REAC protocols in comparison with conventional therapies or placebo as a treatment for reducing symptoms of post-COVID syndrome in adults	Brain stimulation: (1) Neuro-postural optimization (2) Neuro-psycho-physical optimization (3) Restorative tissue optimization	Brazil	05/21–N/A	RCT, double-blinded	TAU and placebo	100	12–18 sessions	QOL, fatigue, cognitive functioning, long COVID symptoms	Breathing, pulmonary tomographic pattern, anxiety, depression, pain perception	N/A	High risk
NCT-05126511 (Koczulla & Schneeberger, 2021)	Effects of cranial electrotherapy stimulation on anxiety of patients after COVID-19	Cranial electrotherapy stimulation	Germany	11/21–03/22	Feasibility/pilot RCT, triple-blinded	Placebo	40	1 h daily, 3 weeks	Anxiety	Insomnia, fatigue, depression, wellbeing, subjective effectiveness, comfort	Baseline, day 21	Some concern^a^
NCT-04944147 (Flöel, 2021)	Cognitive training and brain stimulation in patients with post-COVID-19 cognitive impairment	Anodal transcranial direct current stimulation, cognitive training	Germany	08/21–09/23	RCT, triple-blinded	Placebo	60	2 mA for 20 min, cognitive training, 3 weekly session, 3 weeks	Cognitive functioning	Cognitive functioning, QOL, Post COVID functioning	Baseline, week 3, week 4	Some concern
NCT-04644172 (Taub & McKay, 2020)	Improving thinking in everyday life after COVID-19	Cognitive training in processing speed, activities of daily living, behavioral contract	United States	11/20–11/24	Feasibility/pilot RCT, single-blinded	Waitlist + TAU	40	3.5 h/day, 2–5 days a week, 2–10 weeks	Cognitive functioning, activities of daily living	Cognitive functioning, activities of daily living	Baseline, post intervention, 6 m	High risk^a^
NCT-04843930 (Gunning et al., 2021)	Improving cognitive health in COVID-19 survivors	Cognitive training using an algorithmically delivered video game	United States	06/21–09/22	RCT, double-blinded	Waitlist	125	20–25 min daily, 5–7 days/week, 6 weeks	Cognitive functioning	Daily functioning	Baseline, post intervention	High risk
NCT05338749 (Ownby & Davenport, 2022)	Computer cognitive training for post-acute COVID-19 syndrome	Game-based, computer-delivered cognitive training	United States	04/22–12/23	Feasibility/pilot non-RCT, open label	N/A	10	3 weeks	Intervention usefulness	Cognitive functioning	Baseline, week 3	High risk^a^
NCT05225220 (Zheng et al., 2022)	Multimodal investigation of post-COVID-19 in females	Neuromodulation using transcutaneous vagus nerve stimulation (t-VNS)	United States	03/22–01/23	Feasibility/pilot non-RCT, open label	N/A	20	60 min daily, 10 days	Cognitive functioning	Brain structure, blood marker level, anxiety, depression, sleep quality, fatigue, olfactory performance	Baseline, week 3, week 7	High risk^a^
NCT05212467 (Liira & Arokoski, 2022)	AIR-program and HUS internet therapy compared to treatment as usual in functional disorders and post-COVID-19 condition	Amygdala and insula retraining (AIR): guided self-management program with breathing, meditation, neurolinguistic training	Finland	01/22–12/24	RCT, double-blinded	TAU	360	2 h weekly, 8 weeks	Functional ability	QOL, symptoms, depression, anxiety, sleep, resilience	Baseline, 3 m, 6 m, and 12 m	High risk
Physiotherapy and physical rehabilitation-based interventions												
NCT-04961333 (Bileviciute-Ljungar & Borg, 2021)	Internet-based multidisciplinary rehabilitation for long-term COVID-19 syndrome	Body therapies for breathing, autonomic nervous system, relaxation, mindfulness, aspects of ACT	Sweden	04/21–12/21	RCT, single-blinded	Waitlist	200	Weekly, 8 weeks	QOL, heart rate	Fatigue, breathing, pain, sleep disorder, functioning, activity	Baseline, post intervention, 6 m	High risk
NCT-04935437 (Asimakos & Katsaounou, 2021)	Implementing a rehabilitation program in patients recovering from COVID-19 infection	Physiotherapy rehabilitation (exercise)	Greece	01/21–09/21	RCT, open label	Placebo	40	2x/week, 2 months	QOL, depression, cognitive dysfunction, PTSD, physical health parameters	Body composition and functional status	Baseline, post intervention	High risk
NCT-04898205 (Greenspan et al., 2021)	Cardiopulmonary rehabilitation in COVID-19 long haulers	Treadmill exercise with supplemental oxygen	United States	01/21–01/22	RCT, open label	Placebo	24	1 h session, 2x/week, 12 weeks	Cognition, long COVID-19 symptoms, physical health parameters	Depression, generalized anxiety, state anxiety, trait anxiety, QOL, perception of cognitive function	Baseline, week 4, week 12	High risk
NCT-04572360 (Gao et al., 2020)	Cardiorespiratory exercise and Chinese medicine for rehabilitation of discharged coronavirus disease (COVID-19) patients	Cardiorespiratory exercise and Chinese herbal medicines	Hong Kong	10/20–06/23	RCT, triple-blinded	Waitlist	172	Exercise: 1 h session 2x/day, 3x/week Medicine: 5 g 2x/day 12 weeks	Physical health parameters	Blood biochemistry, QOL, depression, anxiety, loneliness, gut microbiome	Baseline, post intervention, 6 m	Low risk
NCT-04950725 (Wheatley & Shea, 2021)	COVID-19 virtual recovery study	Respiratory muscle training (RMT), nasal breathing	United States	07/21–07/22	RCT, open label	N/A	1500	Twice a day, 4 weeks	Cognitive functioning, long COVID symptoms, physical health parameters	N/A	Baseline, week 2, week 4	High risk
NCT-04647656 (Zilberman-Itskovich, 2020)	Hyperbaric oxygen therapy for post-COVID-19 syndrome	Hyperbaric oxygen therapy (HBOT)	Israel	01/21–01/23	RCT, quadruple-blinded	Placebo	70	90 min, 5 days/week, 8 weeks	Cognitive functioning	QOL, distress, long COVID symptoms, multiple physical health parameters	Baseline, post intervention	Some concern
NCT-05077241 (Nogueira et al., 2021)	Efficacy of home inspiratory muscle training in post-COVID-19 patients: a randomized clinical trial	Inspiratory muscle training	Brazil	08/21–07/23	Feasibility/pilot RCT, double-blinded	Placebo	10	2x/day, 6 weeks	Respiratory muscle strength, dyspnea, and QOL	Cognitive functioning, anxiety, depression, adverse effects, adherence, multiple physical health parameters	Baseline, week 3 week 6, week 12, week 24	Some concern^a^
NCT-05003271 (Sanchez-Ramirez, 2021)	Pulmonary rehabilitation post-COVID-19	Physiotherapy rehabilitation (exercise)	Canada	10/21–07/22	Feasibility/pilot RCT, open label	Placebo	24	45 min, 3 xweek, 8 week	QOL, activities of daily living, fatigue, physical health parameters	N/A	Baseline, week 8	High risk^a^
NCT05218174 (Gilliland & Driver, 2022)	Exercise in adults with post-acute sequelae of SARS-CoV-2 (COVID-19) infection study	Exercise training program and cognitive training using a mobile app	United States	02/22–12/22	RCT, single-blinded	Waitlist	50	1 h/week, 8 weeks	Health and fitness, cognitive functioning, depression	Physiological functioning, dyspnea, level of activity, sleep quality, QOL, anxiety, PTSD, breathlessness, post-traumatic growth, physical health parameters	Baseline, week 11, week 20	High risk
NCT05196529 (Edgell, 2022)	Inspiratory muscle training in ME/CFS and COVID-19 survivors	Inspiratory muscle training	Canada	05/22–01/24	Non-RCT, open label	Control group without long COVID	60	3 times/week, 8 weeks	Physical health parameters, cognitive functioning	Cardiorespiratory fitness, myalgic encephalomyelitis symptoms	Baseline, week 8	High risk
NCT05381675 (Mustafaoğlu & Yasacı, 2022)	Short term results of tele-rehabilitation	Tele-rehabilitation program including aerobic exercises, flexibility exercises, strengthening exercises	Turkey	05/22–09/22	RCT, triple-blinded	TAU	60	2 times/week, 6 weeks	Functional disability due to dyspnea	Pain, sit-to-stand ability, anxiety and depression, sleep quality	Baseline, week 6	Some concern
NCT05172206 (Koczulla & Gloeckl, 2022)	Symptom-based rehabilitation compared to usual care in post-COVID – a randomized controlled trial	Multidisciplinary symptom-based rehabilitation focusing on fatigue, cognition, or physical symptoms	Germany	01/22–02/23	RCT, single-blinded	TAU	132	3 weeks	QOL	Long COVID symptoms, exercise performance, health care service needs, working capability, sleep quality, depression, anxiety, resilience, cognitive functioning, physical health parameters	Baseline, week 4, week 12	High risk

QOL, quality of life; PTSD, post-traumatic stress disorder; TAU, treatment as usual; N/A, not available; RCT, randomized-controlled trial; ACT, acceptance and commitment therapy; CBT, cognitive-behavioral therapy; m, month.

a These are feasibility or pilot trials not intended to produce complete unbiased outcomes.

The trials are targeting a total target sample size of 5814 participants (median: 65; range: 10–1500). The trials are geographically distributed across 14 countries. Most are described as either randomized efficacy controlled trials (23, 54.8%) or randomized-controlled feasibility/pilot trials (12, 28.6%). All trials are being conducted among adults, and the majority are also including geriatric populations. Trials list an average of 2.8 primary outcomes (s.d. = 3.0, range 1–12), which are being measured using an average of 3.5 primary outcome measures (s.d. = 4.8, range = 1–21). They list an additional average of 5.0 secondary outcomes (s.d. = 3.6, range 0–14) and 6.1 secondary outcome measures (s.d. = 5.1, range 0–23). Nine (21.4%) identified mental health (i.e. depression, anxiety, distress) as among their primary outcomes, while 18 (42.9%) listed cognition, and 17 (40.5%) listed psychological wellbeing.

### Psychological interventions

Eleven trials are examining psychological interventions: five self-management programs (Blázquez, 2022; Busse & Potter, 2022; Chantal, Hiyam, & Karen, 2021; Collette, Willems, Cabello, & Lesoinne, 2022; Culos-Reed & Twomey, 2021; Martin & Lynall, 2022), three cognitive-behavioral therapy interventions (Acartürk & Öztürk, 2021; Håkansson, Hartman, & Cronhjort, 2021; Martin & Lynall, 2022) (one with components of acceptance and commitment therapy and one using peer support), two meditation interventions (Croghan, Hurt, Fokken, & Currie, 2022; Subramaniam, 2021), one psychoeducational intervention (Collette et al., 2022), and one case-management intervention (Hatcher, Ward, & Edgar, 2021). Components of multidisciplinary care are present in several; for example, a cognitive-behavioral therapy intervention includes stretching exercises (Acartürk & Öztürk, 2021), while other interventions include psychoeducation across cognition, diet, breathing, and other spheres of life (Blázquez, 2022; Busse & Potter, 2022; Culos-Reed & Twomey, 2021; Martin & Lynall, 2022). However, none are fully integrated models of care. Intervention durations range from 3 weeks to 3 months. Eight trials are randomized, including three randomized-controlled feasibility or pilot trials. The interventions target primary and secondary outcomes such as depression, anxiety, distress, self-efficacy, cognition, and quality of life, as well as study feasibility variables and other long COVID symptoms. Two have mental health-specific inclusion criteria, i.e. clinical distress or a positive screen for clinical depression or anxiety (Acartürk & Öztürk, 2021; Håkansson et al., 2021), another includes cognitive impairment as an inclusion criterion (Collette et al., 2022), and one lists quality-of-life impairments as a requirement to participate (Hatcher et al., 2021). Six trials exclude individuals with severe or acute mental illness, or a history of mental illness (Acartürk & Öztürk, 2021; Blázquez, 2022; Collette et al., 2022; Croghan et al., 2022; Håkansson et al., 2021; Subramaniam, 2021), and two exclude individuals with substance use disorders (Acartürk & Öztürk, 2021; Collette et al., 2022); severe cognitive deficits are an exclusion criterion for three trials (Collette et al., 2022; Hatcher et al., 2021; Håkansson et al., 2021).

### Pharmacological and other medical interventions

Five interventions are testing pharmacological and other medical treatments for long COVID. Pharmacological agents include the selective serotonin reuptake inhibitor vortioxetine (McIntyre & Subramaniapillai, 2021), a C1 esterase inhibitor (recombinant) (Melamed, Collins, & Palm, 2021), and atorvastatin (Anderson & Carcel, 2021). Other medical treatments include portable oxygen concentrator (Glezer, 2022), and a one-time marrow stromal cell infusion (Alderman, Montemayor, & Savitz, 2022). The trials focus on improving cognitive functioning, mood, or functioning more broadly. None describe integrated models of care. Intervention duration ranges from one-time treatment to 6 months. All five trials are randomized and the majority are at least partially blinded, and three are placebo controlled (Alderman et al., 2022; McIntyre & Subramaniapillai, 2021; Melamed et al., 2021). None of the studies have any mental health or wellbeing-specific inclusion criteria. Two trials require self-reported cognitive deficits to participate (Glezer, 2022; McIntyre & Subramaniapillai, 2021). Severe mental illness and dementia are among the exclusion criteria for three (Anderson & Carcel, 2021; McIntyre & Subramaniapillai, 2021); one excludes individuals with substance use disorders (McIntyre & Subramaniapillai, 2021).

### Herbal, nutritional, or natural supplement interventions

Five trials are investigating herbal, nutritional, or natural supplements. These trials are diverse, examining cannabidiol-dominant medicinal cannabis (Iveson, Lynskey, & Thurgur, 2021), a dietary replacement and weight management program (Blane, Combet, & the ReDIRECT Study Team, 2021), niagen (vitamin B3) (Guzman-Velez, Gutiérrez-Martínez, González-Irizarry, & Gerber, 2021), a homeopathic medicinal combination (Rice & Jacobs, 2021), and a mixed herbal supplement (Karosanidze & Panossian, 2021). A sixth trial is examining a Chinese herbal medicine intervention integrated with physical rehabilitation (Gao et al., 2020), which is described in the physical rehabilitation section below. The other five trials are not described as examining integrated models of care.

Intervention duration ranges from 2 weeks to 5–6 months in the four of five studies reporting a duration. Four of five trials are randomized, three with placebo control groups and one with a waitlist control group. The medicinal cannabis study is a single-group pilot trial (Iveson et al., 2021). Primary and secondary outcomes include depression and anxiety, cognitive function, quality of life, fatigue, long COVID symptoms in general, and a variety of physical health metrics. None of the studies require that individuals have impairments to mental health or wellbeing to participate, but one study requires cognitive deficits (Guzman-Velez et al., 2021). Four trials list severe, chronic, or pre-existing mental illness as an exclusion criterion (Blane et al., 2021; Guzman-Velez et al., 2021; Iveson et al., 2021; Rice & Jacobs, 2021), and three exclude individuals with substance use disorders (Guzman-Velez et al., 2021; Iveson et al., 2021; Rice & Jacobs, 2021). None list any cognitive impairment factors as exclusion criteria.

### Cognitive and neurorehabilitation interventions

Nine trials are examining cognitive and neurorehabilitation interventions, using cognitive training or brain stimulation. Five interventions focus on cognitive rehabilitation therapies with diverse approaches and techniques, from using digital devices (Flöel, 2021; Gunning, Oberlin, & Victoria, 2021; Ownby & Davenport, 2022; Taub & McKay, 2020), adapting elements of cognitive therapy (Taub & McKay, 2020), or incorporating breathing, meditation, and neurolinguistic programming (Liira & Arokoski, 2022). Four trials are examining the effectiveness of neuromodulation and neurostimulation technologies such as transcranial direct current stimulation, cranial electrotherapy stimulation, or transcutaneous vagus nerve stimulation (Eryilmaz, Andreou, & Pax, 2021; Koczulla & Schneeberger, 2021; Neri & Barcessat, 2021; Zheng, Wang, & Fullmer, 2022). One trial combines brain stimulation and intensive cognitive training (Flöel, 2021); however, none of the trials indicate integrated, multidisciplinary care. Intervention length ranges from 10 days to 10 weeks. The majority of the studies are randomized-controlled trials with placebo or waitlist control groups receiving sham stimulation or treatment as usual. Two out of nine trials are feasibility non-randomized studies with no comparison group (Ownby & Davenport, 2022; Zheng et al., 2022). For six trials, primary outcomes focus on cognitive function; one trial identifies anxiety as the primary outcome (Koczulla & Schneeberger, 2021). Other primary and secondary outcomes include quality of life, fatigue, daily functioning, depression, and other symptoms associated with long COVID. Six of the nine trials require some degree of cognitive impairment to participate (Eryilmaz et al., 2021; Flöel, 2021; Gunning et al., 2021; Ownby & Davenport, 2022; Taub & McKay, 2020; Zheng et al., 2022) and one lists anxiety as an inclusion criterion (Koczulla & Schneeberger, 2021). Four exclude individuals with pre-existing severe cognitive impairment (Flöel, 2021; Gunning et al., 2021; Ownby & Davenport, 2022; Taub & McKay, 2020). Six exclude those with mental illness or substance use disorders (Eryilmaz et al., 2021; Flöel, 2021; Gunning et al., 2021; Liira & Arokoski, 2022; Ownby & Davenport, 2022; Taub & McKay, 2020).

### Physiotherapy and physical rehabilitation-based interventions

Twelve trials are examining physiotherapy or physical rehabilitation-based interventions, focusing on respiratory or cardio-respiratory rehabilitation (Bileviciute-Ljungar & Borg, 2021; Edgell, 2022; Gao et al., 2020; Greenspan et al., 2021; Nogueira, Silva, & Nogueira, 2021; Sanchez-Ramirez, 2021; Wheatley & Shea, 2021), exercise and strength training (Asimakos & Katsaounou, 2021; Gao et al., 2020; Gilliland & Driver, 2022; Mustafaoğlu & Yasacı, 2022; Sanchez-Ramirez, 2021), hyperbaric oxygen therapy (Zilberman-Itskovich, 2020), and symptom cluster-based rehabilitation (Koczulla & Gloeckl, 2022). Some multidisciplinary integration of treatments is reported. One trial includes mindfulness, relaxation, and psychotherapeutic components (Bileviciute-Ljungar & Borg, 2021), another refers to psychological and dietary supports (Asimakos & Katsaounou, 2021), while one study combined individualized exercises with cognitive training (Gilliland & Driver, 2022). One trial integrates cardiorespiratory rehabilitation with a combination of Chinese herbal medicines (Gao et al., 2020). Intervention duration ranges from 3 to 12 weeks. Most of the studies are randomized-controlled trials, with sham treatment, waitlists, or active control groups. Primary and secondary outcomes are varied, including depression, anxiety, cognition, quality of life, and a range of physiological and functional outcomes. None of the 12 trials list any mental health variables as inclusion criteria; one requires cognitive deficits (Zilberman-Itskovich, 2020) and two require negative impacts on quality of life (Asimakos & Katsaounou, 2021; Zilberman-Itskovich, 2020). Two trials exclude individuals with severe cognitive deficits or dementia (Asimakos & Katsaounou, 2021; Sanchez-Ramirez, 2021), and two exclude those with substance use disorders (Bileviciute-Ljungar & Borg, 2021; Zilberman-Itskovich, 2020). Three exclude individuals with mental illness (Bileviciute-Ljungar & Borg, 2021; Gao et al., 2020; Sanchez-Ramirez, 2021), two only if the mental illness is untreated or uncontrolled (Bileviciute-Ljungar & Borg, 2021; Gao et al., 2020).

### Limited risk-of-bias assessment

Overall risk-of-bias assessment is reported in Table 2.

#### Randomization

Most of the registered trials report randomization (35, 83.3%), a low risk-of-bias indicator. However, only four records confirm that they will implement allocation concealment (9.5%); information about the randomization process is missing for all other records.

#### Masking

Fifteen studies (35.7%) report that participants and/or treatment providers are masked, lowering the risk of bias.

#### Outcome measurement

Nineteen trials (45.2%) achieved a low risk-of-bias rating, with appropriate masked measurement processes that would not be expected to be influenced by bias. Four trials (9.5%) are associated with some concern, and 19 trials (45.2%) have a high risk-of-bias rating, generally due to open label designs and possible interviewer or self-report biases in outcome assessments.

## Discussion

Given the rapid emergence and global spread of COVID-19, it has taken time to move from identifying long COVID to testing treatments for it. A small international body of research is assessing interventions for mental health, cognition, and psychological wellbeing in long COVID. Several psychological interventions are being tested, but few full-scale psychotherapeutic interventions are being trialed to date. Only a few interventions with pharmacological and other medical treatments were found, complemented by a similar number of herbal, nutritional, or natural supplement interventions. Several physical and cognitive rehabilitation interventions are also being examined. Randomized-controlled trials and randomized-controlled feasibility trials dominate the trial landscape.

We laud researchers who have quickly registered trials and begun testing interventions for this new clinical entity. At the same time, we highlight that the number, size, and quality of trials and the breadth of interventions are limited. Given the potential long-term disability associated with long COVID (Alwan, 2021; Brown & O'Brien, 2021), we call on funders to support research in this area at a level commensurate with symptomatic burden. We also call on interventionists to rapidly pursue large-scale, rigorous, high-quality clinical trials on interventions that address the full range of long COVID symptoms, including mental health, cognition, and psychological wellbeing (Crook et al., 2021; Malik et al., 2022; Maury et al., 2020; Sudre et al., 2021). Adaptive trials may be the most promising design approach to address the mental health symptoms of long COVID, in the context of an evolving pandemic and emerging knowledge base (Janiaud, Hemkens, & Ioannidis, 2021). Given the urgent need to build a new evidence base, juxtaposed with typically high rates of non-publication of clinical trials (Lee, Bacchetti, & Sim, 2008), researchers are encouraged to publish their findings – positive or negative – at the earliest possible date (Mlinaric, Horvat, & Supak Smolcic, 2017). Likewise, publishers and peer reviewers are encouraged to welcome both positive and negative findings to accelerate the construction of a balanced and comprehensive evidence base in this new domain.

NICE guidelines recommend integrated, interdisciplinary treatments for long COVID (International Foundation of Integrated Care, 2020), but the current trials demonstrate limited service integration. Integrated, interdisciplinary models of care that directly address a broad range of symptoms are needed, and they should be rigorously evaluated using methodologies appropriate for complex interventions (Skivington et al., 2021). Many of the registered trials are broadly scanning for outcomes in an integrated manner, across biological and psychological spheres, which is also important to continue. The ongoing use of virtual service features will provide important advancements for the evidence base on virtual healthcare interventions (Torous, Jän Myrick, Rauseo-Ricupero, & Firth, 2020).

While many of trials currently registered are addressing mental health in some way, comparatively few trials focus explicitly on mental health, *v.* cognition and psychological wellbeing. Importantly, interventions targeting individuals with severe mental illness or pre-existing mental illness are absent, and a number of trials explicitly identify mental illness as an exclusion criterion. Not only can long COVID be associated with the emergence of new mental health challenges (Aiyegbusi et al., 2021), but some long COVID patients will have pre-existing mental illness, which is a risk factor for long COVID (Gebhard et al., 2021) and may affect the experience of long COVID. Similarly, substance misuse is a very common comorbidity among people with mental illness (Lai, Cleary, Sitharthan, & Hunt, 2015), yet none of the registered trials mentioned substance misuse, except as an exclusion criterion. We therefore call on interventionists to develop and evaluate interventions that integrate evidence-based treatments for mental illness and substance misuse with treatments for the physiological symptoms of long COVID, while also addressing the potential interaction between mental and physical health.

Given the novelty of this clinical entity, it is unsurprising that trials are recruiting from the general population of patients with long COVID. A next, critical step is to test interventions adapted to vulnerable subpopulations. With a focus on equity, diversity, and inclusion, interventions should attend to individuals with different sociodemographic characteristics, including youth and seniors, and subgroups of people who are facing challenges with various social determinants of health, physical and mental health comorbidities, limited access to digital technologies, and other treatment access barriers. While doing so, attending to generalizability within interventions and trial designs may provide gains for other disorders with overlapping symptomatology (Wong & Weitzer, 2021). Researchers are encouraged to reflect on additional knowledge gaps and opportunities, from their unique disciplinary perspectives, and to move forward with addressing them in a timely manner.

We further call on the research community to engage patients in the research and service design process to address long COVID, from a pragmatic, patient-oriented research perspective (Allemang, Sitter, & Dimitropoulos, 2022; Canadian Institutes of Health Research, 2019). Only two of the registered trials refer to patient-engaged research processes (Busse & Potter, 2022; Martin & Lynall, 2022). However, patients first identified long COVID as a clinical entity (Callard & Perego, 2021), demonstrating their important insights into their lived experience and their ability to advocate for themselves to drive change. Through co-creation, patients can make meaningful contributions to research and service design (Canadian Institutes of Health Research, 2019; Hamilton et al., 2018).

This review has a number of limitations. Notably, the pace of COVID-19 research is extremely rapid (Liu et al., 2021b). This review is limited to trials registered by 31 May 2022; any trials registered after this date, or not registered, are not included. Given the limited amount of information available in trial registries, only a partial quality appraisal was possible. Due to the lack of trial results to date, a meta-analytical report was not possible. Researchers are encouraged to register their trials, consult the trial registries for studies aligning with their area of work, and report their results rapidly to members of the scientific and clinical care communities, many of whom are eagerly awaiting their findings.

## Conclusions

An emerging body of research has begun to test interventions for mental health, cognition, and psychological wellbeing in long COVID. However, this review highlights that the scope of the associated intervention research currently in progress is not yet commensurate with the scope of this important new clinical entity. Despite a great deal of uncertainty around the evolution of long COVID, it is incumbent on researchers to build upon the trials currently under way and to rapidly generate rigorous evidence in this entirely new domain. We therefore call on researchers around the world to develop high-quality clinical trials testing a wide range of candidate interventions addressing mental health, cognition, and psychological wellbeing in diverse patient populations experiencing the symptoms of long COVID.
